# A Japanese case of castration-resistant prostate cancer with *BRCA2* and *RB1* co-loss and *TP53* mutation: a case report

**DOI:** 10.1186/s12920-022-01286-w

**Published:** 2022-06-20

**Authors:** Tomohiro Iwasawa, Takeo Kosaka, Shinya Morita, Shuji Mikami, Kohei Nakamura, Hiroshi Hongo, Hiroshi Nishihara, Mototsugu Oya

**Affiliations:** 1 Department of Urology, Keio University School of Medicine, 35 Shinanomachi, Shinjuku-ku, Tokyo, 160-8582 Japan; 2 Genomics Unit, Keio Cancer Center, Keio University School of Medicine, 35 Shinanomachi, Shinjuku-ku, Tokyo, 160-8582 Japan; 3 Department of Pathology, Keio University School of Medicine, 35 Shinanomachi, Shinjuku-ku, Tokyo, 160-8582 Japan

**Keywords:** Castration-resistant prostate cancer, Genomic profiling, *RB1*, *BRCA2*, *TP53*

## Abstract

**Background:**

Abnormalities in homologous recombination contribute to the aggressive nature of castration-resistant prostate cancer. Retinoblastoma transcriptional corepressor 1 (*RB1*) and breast cancer 2 (*BRCA2*) exist close to each other in the same chromosome, and the significance of their concurrent loss has become a hot topic in the field of cancer research.

**Case presentation:**

A 61-year-old man presented with a chief complaint of a mass on his head and was diagnosed as multiple bone metastases from prostate cancer. He was treated with standard medication, but he died 2 years 6 months after being diagnosed with prostate cancer. Simultaneous biallelic loss of *RB1* and *BRCA2* as well as a truncating mutation of tumor protein p53 (*TP53*) were revealed by genomic analysis.

**Conclusion:**

To our knowledge, this is the first report of castration-resistant prostate cancer (CRPC) with *BRCA2* and *RB1* co-loss and *TP53* mutation. To establish a treatment strategy for highly malignant cases with such multiple genetic features is important.

**Supplementary Information:**

The online version contains supplementary material available at 10.1186/s12920-022-01286-w.

## Background

Prostate cancer is the most common type of cancer among men in the United States, and its prevalence is increasing in Japan [1]. The standard treatment for advanced prostate cancer is androgen deprivation therapy (ADT), but various mechanisms result in resistance to this therapy, leading to castration-resistant prostate cancer (CRPC). Enzalutamide, abiraterone, and cabazitaxel are currently available as therapeutic agents for metastatic CRPC, and treatment strategies have dramatically changed over time. Abnormalities in homologous recombination (HR) contribute to the aggressive nature of CRPC [2]. Olaparib, a poly(ADP-ribose) polymerase (PARP) inhibitor that has just been approved for use in Japan, was shown to be effective for CRPC with breast cancer 1, breast cancer 2 (*BRCA2*), or ATM serine/threonine kinase mutations in the PROfound trial [3]. However, the detailed clinicopathological features of CRPC with HR defects are still unclear. Herein, we report on the case of a Japanese patient with CRPC in whom co-loss of retinoblastoma transcriptional corepressor 1 (*RB1*) and *BRCA2* as well as a mutation of tumor protein p53 (*TP53*) were detected.

## Case presentation

**Fig. 1:**
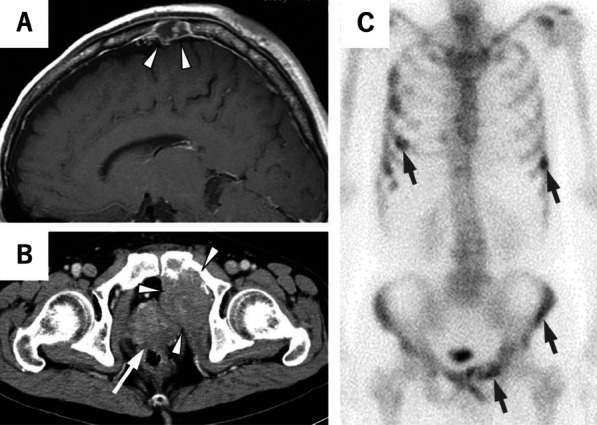
Imaging at diagnosis. **A** Magnetic resonance image of a tumor in the patient’s skull. **B** Computed tomography scan image showing osteolytic lesions on the pubis, adjacent to the abnormally enlarged prostate. **C** ^99m^Tc-Hydroxymethylene diphosphonate bone scintigraphy

A previous healthy 61-year-old man visited a local doctor with a chief complaint of a mass on his head. He was referred to the neurosurgery department of our hospital on suspicion of a tumor in the skull. Magnetic resonance imaging of the head was performed by 3 T device (GE DISCOVERY MR750; GE Healthcare) with Gadolinium-based contrast agent, and metastatic tumor was suspected (Fig. 1A). Blood examination showed a high prostate-specific antigen level of 165.42 ng/mL, and a computed tomography scan detected lytic lesions of pelvic bone adjacent to the abnormally enlarged prostate. (Fig. 1B). ^99m^Tc-Hydroxymethylene diphosphonate bone scintigraphy revealed multiple bone metastases throughout the body (Fig. 1C). The patient was further referred to our department on suspicion of multiple bone metastases from prostate cancer.

**Fig. 2:**
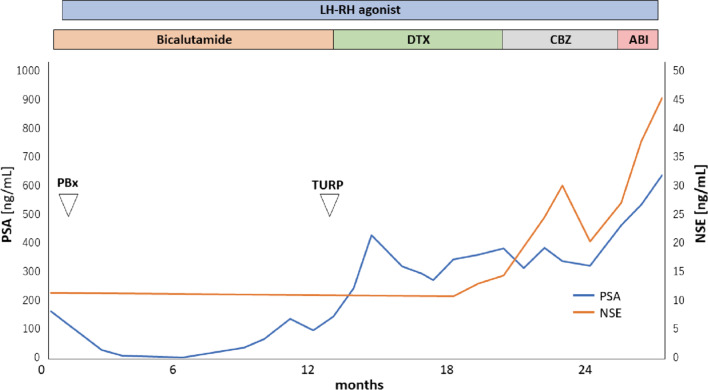
Treatment course of the patient. Abbreviations: ABI, abiraterone acetate; CBZ, cabazitaxel; DTX, docetaxel; LH-RH, luteinizing hormone–releasing hormone; NSE, neuron-specific enolase; PBx, prostate biopsy; PSA, prostate-specific antigen; TURP, transurethral resection of the prostate

A needle biopsy of the patient’s prostate revealed an adenocarcinoma with a Gleason score of 5 + 4 = 9, which prompted ADT. Elevation of the prostate-specific antigen level and postrenal failure due to urinary retention were observed 1 year after ADT started, and channel transurethral resection of the prostate (TURP) was thus performed. Docetaxel was administered for a total of seven courses because the patient’s tumor was considered to have acquired castration resistance. However, multiple lymph node and bone metastases were exacerbated. Although a total of six courses of cabazitaxel had already been administered, it was discontinued owing to sacral pressure ulcer infection and deterioration of the patient’s general condition. He was subsequently treated with abiraterone, but he died 2 years 6 months after being diagnosed with prostate cancer (Fig. 2).

We performed genetic sequencing of 160 cancer-related genes (PleSSision-Rapid®) using specimens collected by TURP. *RB1* and *BRCA2* co-deletion as well as a truncating mutation of *TP53* (G244Rfs*19) were detected. Amplification of the androgen receptor (*AR*) gene was observed with an estimated copy number of 18.3. In addition, a patched 1 (*PTCH1*) point mutation (p.R441H) was detected as a possibly pathogenic alteration.

Immunohistochemical staining was performed using standard protocols. The products of the antibodies and the detailed protocols are shown in the additional file. All stained sections were scanned using a high-resolution digital slide scanner (NanoZoomer-XR C12000; Hamamatsu Photonics, Hamamatsu, Shizuoka, Japan), which consisted of a trilinear sensor camera, a detector of 4096 pixels × 64 lines x 3plates, and a filter that only divides into RGB with a prism. The measured resolution of all microscopy images was 0.23 μm/pixel, which was equivalent to 40 × objective lens.

**Fig. 3:**
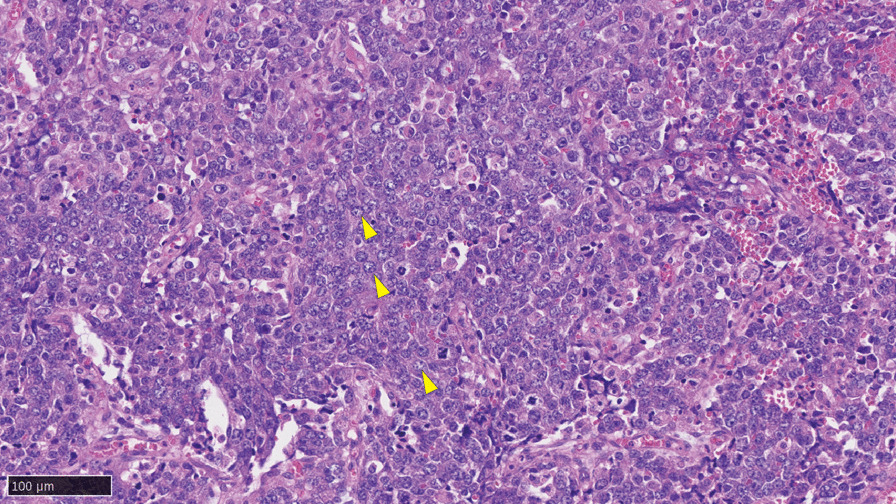
Hematoxylin and eosin staining of transurethral resection sample. Yellow arrows indicate tumor cells with clear nucleoli within a large nucleus. The black bar shows 100 μm

Hematoxylin and eosin staining showed that tumor cells had clear nucleoli and a histology different from that of typical neuroendocrine prostate cancer cells (Fig. 3). Complete absence of RB1 and p53 protein expressions (Additional file 1: Fig. S1A–B) were consistent with genomic findings. AR was stained in 70% of tumor nuclei and PSA was positive in about 30% of tumor cells (Additional file 1: Fig. S1C–D), whereas neuroendocrine markers were not stained (Additional file 1: Fig. S1E–G).

Immunohistochemical staining of glioma-associated oncogene family zinc finger 1 (GLI1) was performed to confirm whether the hedgehog signal was enhanced by a mutation of *PTCH1* in our case. GLI1-positive tumor cells accounted for less than 10% of the total (Additional file 1: Fig. S1H) and the nuclear stainability of the cells was not stronger than that of Leydig cells used as positive control (data not shown), indicating that the hedgehog signal was not enhanced.

## Discussion and conclusions

The case herein described is characterized by (1) Co-loss of *RB1* and *BRCA2*, (2) *TP53* mutation, (3) *PTCH1* mutation, and (4) A poorly differentiated AR-positive cancer without neuroendocrine features. Chakraborty et al.[4] demonstrated that concurrent loss of *RB1* and *BRCA2* leads to resistance to ADT and acquisition of epithelial-mesenchymal transition. Both *RB1* and *BRCA2* exist in the long arm of chromosome 13; even with a single-copy loss, co-loss of *RB1* and *BRCA2* is associated with shortened survival and increased genomic mutations. The homodeletion of both genes in our case could have contributed to the aggressive clinical course and treatment resistance observed. In addition, the impaired HR due to the BRCA2 deletion could account for the large fluctuation in gene copy numbers.

**Fig. 4:**
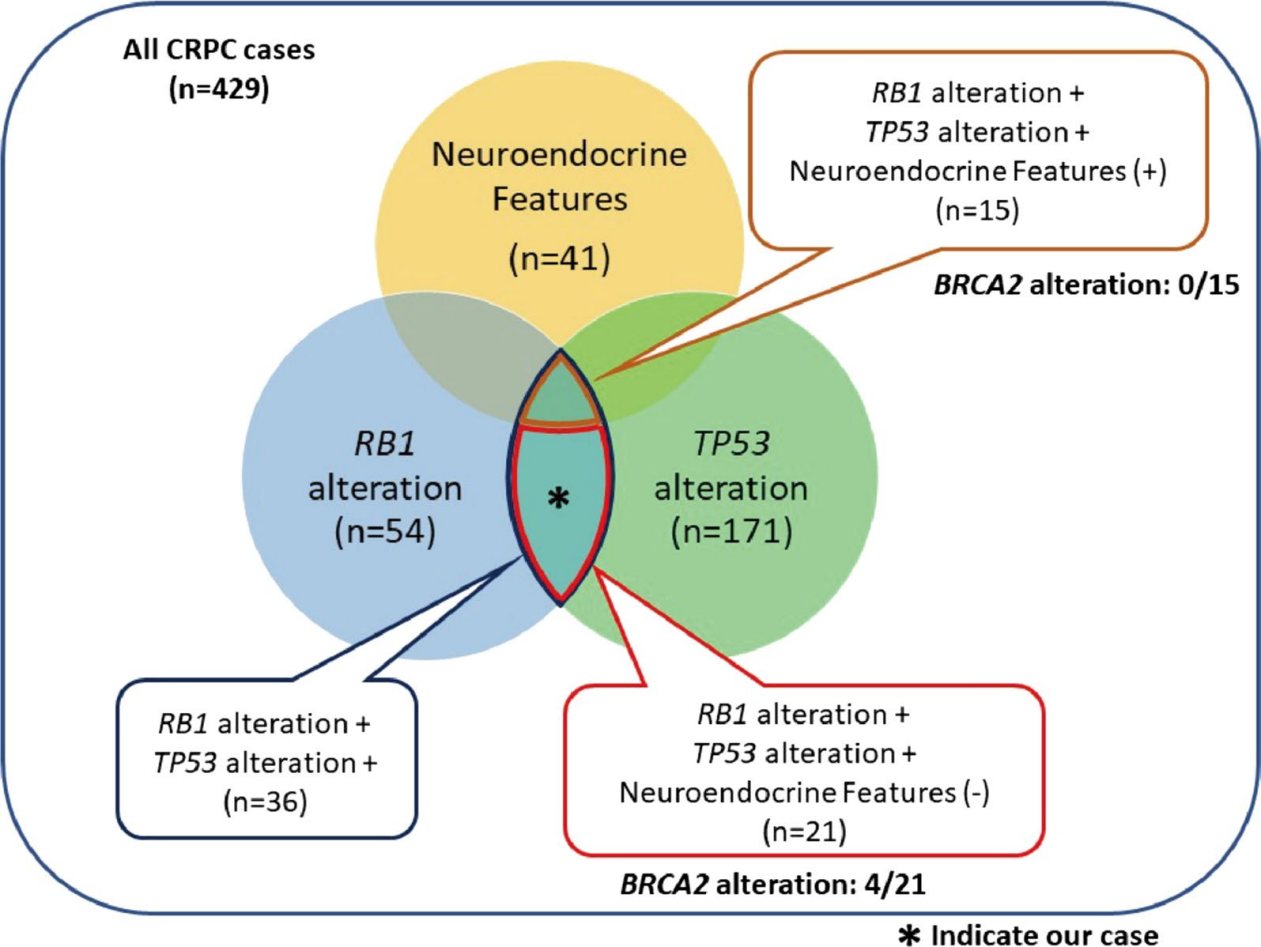
Results of analyzing the database of prostate cancer patients using cBioportal. Analysis of the data of the Stand Up To Cancer–Prostate Cancer Foundation Prostate Dream Team using cBioPortal. Our case is marked by an asterisk. Abbreviations: *BRCA2*, breast cancer 2; CRPC, castration-resistant prostate cancer; *RB1*, retinoblastoma transcriptional corepressor 1; *TP53*, tumor protein p53

The concurrent loss of *TP53* and *RB1*, which are well-known tumor suppressor genes, is one of the genomic features of neuroendocrine prostate cancer [5]. Ku et al.[6] reported that knockdown of *TP53* and *RB1* in prostate adenocarcinoma caused neuroendocrine differentiation, resulting in decreased *AR* expression and increased neuroendocrine marker expression. Analysis of the data of the Stand Up To Cancer–Prostate Cancer Foundation (SU2C/PCF) Prostate Dream Team[7] using cBioPortal, which is the largest database for metastatic CRPC, revealed that 36 of 429 cases exhibited gene alterations in both *TP53* and *RB1* but that only 15 cases exhibited neuroendocrine differentiation (Fig. 4). *BRCA2* alteration was found only in the non-neuroendocrine group in both *TP53* and *RB1* altered cases. In our case, both TP53 and RB1 were not stained, but the neuroendocrine markers were negative and AR was positive. The possibility that our patient’s adenocarcinoma was in the process of neuroendocrine differentiation at the time of TURP could not be excluded.

PTCH1 is a major component of hedgehog signaling and regulates the glioma-associated oncogene family of downstream transcription factors, and it is reported to be involved in the development or progression of prostate cancer [8]. In the COSMIC database, of a total of 2803 prostate cancer samples, 52 (1.86%) had *PTCH1* mutations. The same *PTCH1* R441H mutation detected in our case has been reported in cases of breast cancer and colorectal cancer, but the clinical significance of both cases is unknown [9, 10]. The results of immunohistochemical staining did not show activation of GLI1, a transcription factor downstream of PTCH1, suggesting that the *PTCH1* missense mutation seen in our case might have been a passenger mutation.

The National Comprehensive Cancer Network[11] guidelines recommend a platinum-based regimen for only CRPC that presents pathologic neuroendocrine differentiation. However, non-neuroendocrine CRPC cases harboring genetic neuroendocrine prostate cancer features, such as *RB1* loss and *TP53* mutation, could be in the process of neuroendocrine differentiation such that they could have already acquired sensitivity to platinum-based chemotherapy. Corn et al.[12] reported that the combination of carboplatin and cabazitaxel was effective for the aggressive variant prostate cancer molecular signature with defects in at least two of the three tumor suppressor genes, namely, *RB1*, *TP53*, and *PTEN*. In addition, research has shown that sensitivity to platinum-based chemotherapy is higher in CRPC patients with a DNA repair gene aberration, such as breast cancer 1, *BRCA2*, or ATM serine/threonine kinase, than in those without it [13]. These data suggest that platinum-based chemotherapy and PARP inhibitor treatment could be effective in our case. Further analysis is needed to evaluate whether currently available PARP inhibitors would be just as effective in cases with other genetic features.

To our knowledge, this is the first report of CRPC with *BRCA2* and *RB1* co-loss and *TP53* mutation. Because it is unclear whether therapeutic strategies targeting specific genetic changes are effective, to establish a treatment strategy for highly malignant cases with such multiple genetic features by accumulating cases is important.

## Supplementary Information

**Additional file1.** Immunohistochemical staining of transurethral resection samples. (A) Tumor protein p53. (B) Retinoblastoma transcriptional corepressor. (C) Androgen receptor. (D) Prostate-specific antigen. (E) Synaptophysin. (F) Chromogranin A. (G) Cluster of differentiation 56. (H) Glioma-associated oncogene family zinc finger 1. The bars show 100μm**.**

## Data Availability

The genome data generated and analyzed during the current study have been deposited in the Genome Sequence Archive for Human repository under accession code HRA002421. The data are available from the following link: https://ngdc.cncb.ac.cn/gsa-human/browse/HRA002421

## Abbreviations

*RB1*: Retinoblastoma transcriptional corepressor 1
*BRCA2*: Breast cancer 2
*TP53*: Tumor protein p53
*AR*: Androgen receptor
*PTCH1*: Patched 1
*GLI1*: Glioma-associated oncogene family zinc finger 1
PARP: Poly(ADP-ribose) polymerase
ADT: Androgen deprivation therapy
CRPC: Castration-resistant prostate cancer
HR: Homologous recombination
TURP: Transurethral resection of the prostate
