# Supplementary material for: A Japanese case of castration-resistant prostate cancer with BRCA2 and RB1 co-loss and TP53 mutation: a case report
Source: BMC Med Genomics. 2022 Jun 20;15:138. doi: 10.1186/s12920-022-01286-w (PMC9208097; doi:10.1186/s12920-022-01286-w)

(A)
Tumor protein p53 (p53)

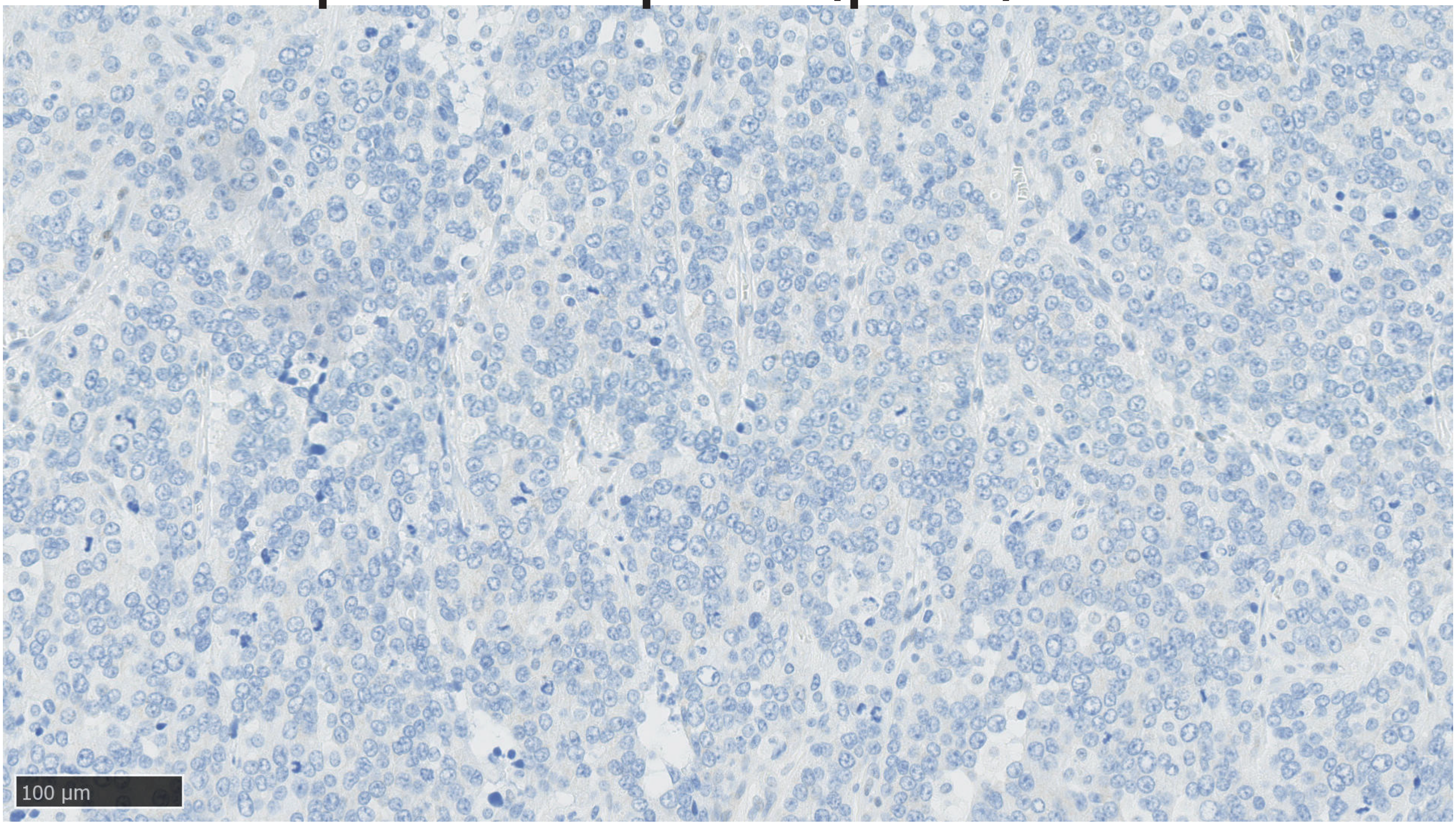

(B)
Retinoblastoma protein (RB1)

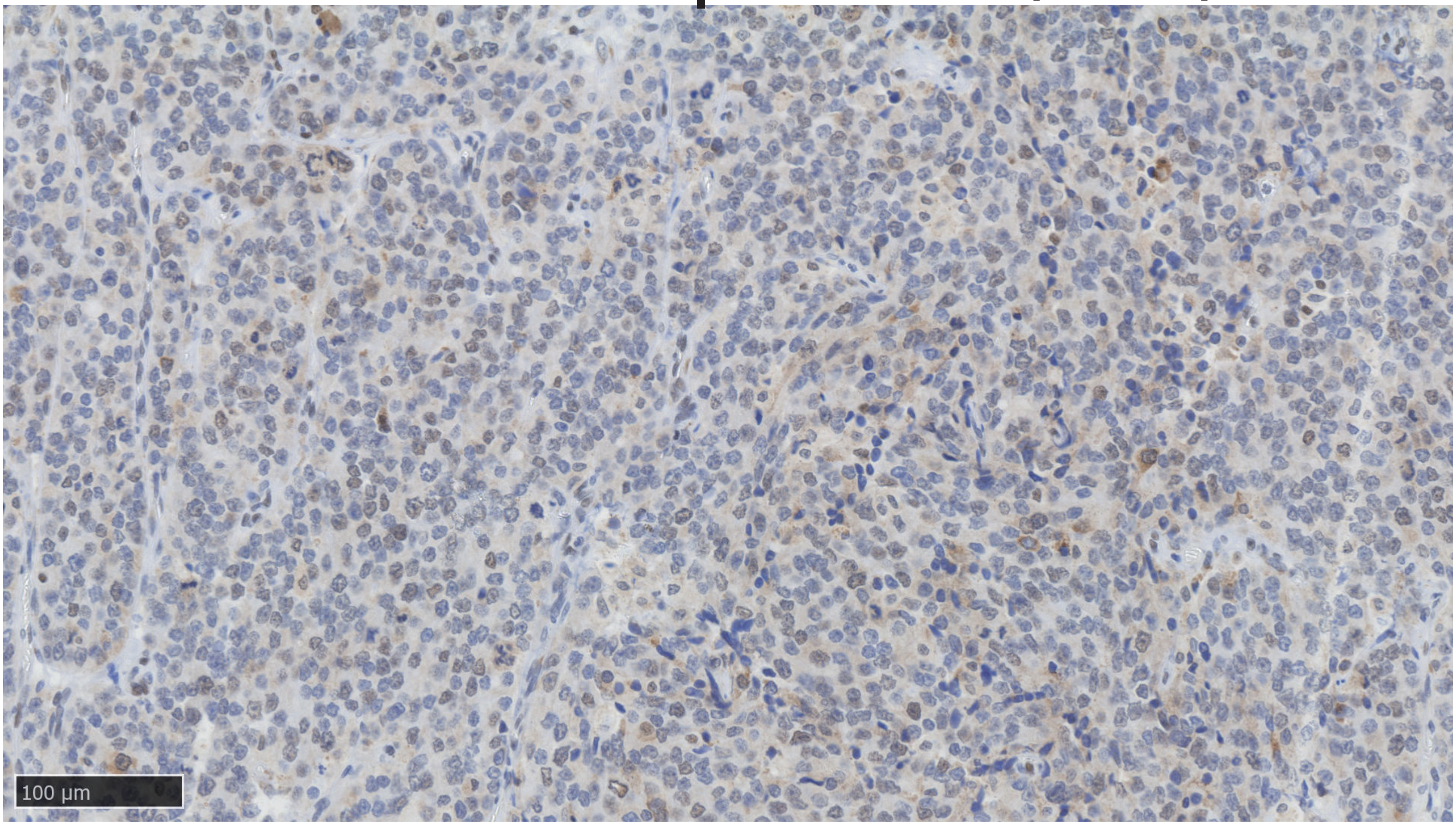

(C)
Androgen receptor (AR)

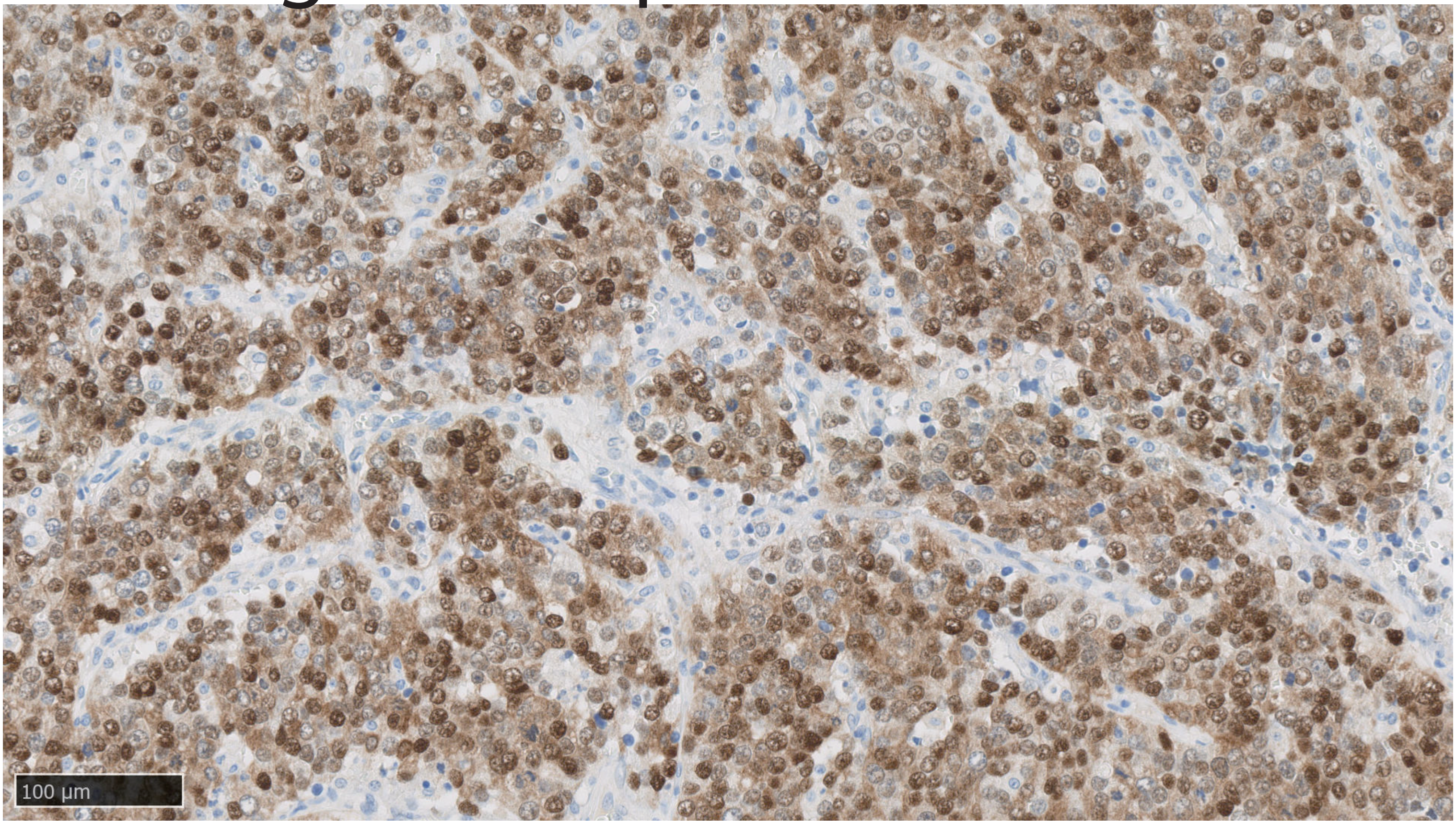

(D)
Prostate specific antigen (PSA)

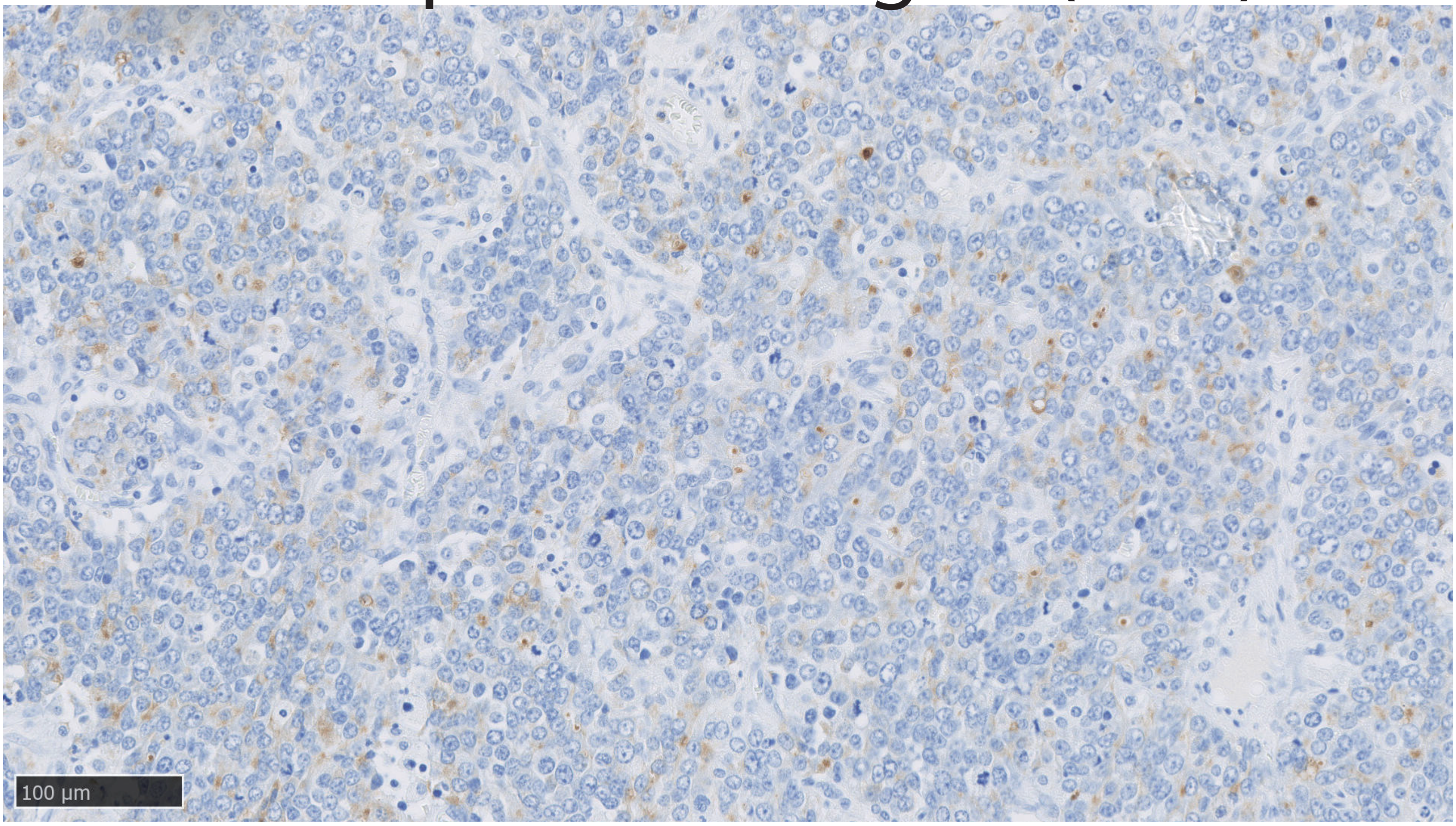

(E)
Synaptophysin (SYP)

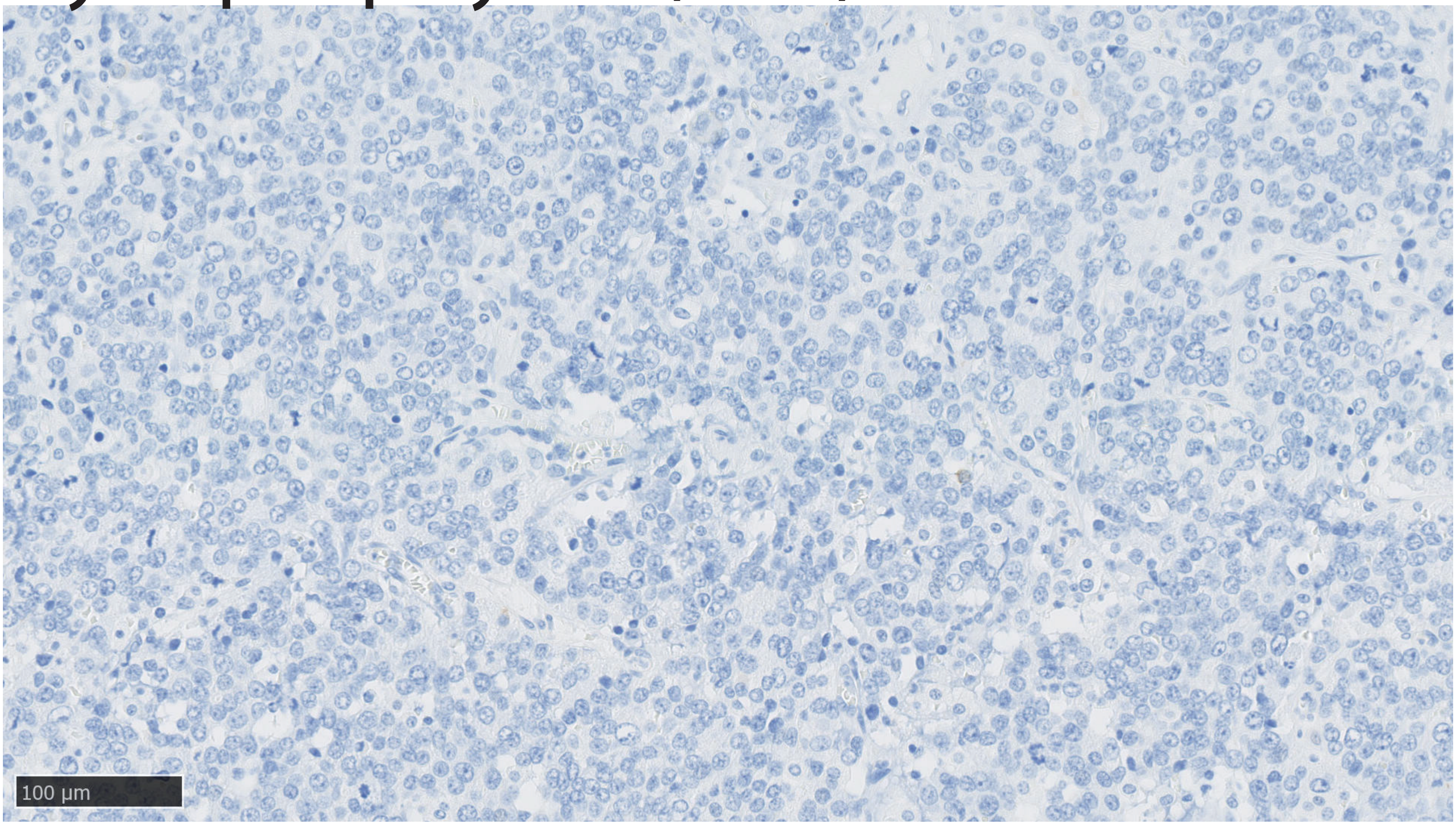

(F)
Chromogranin A (CHGA)

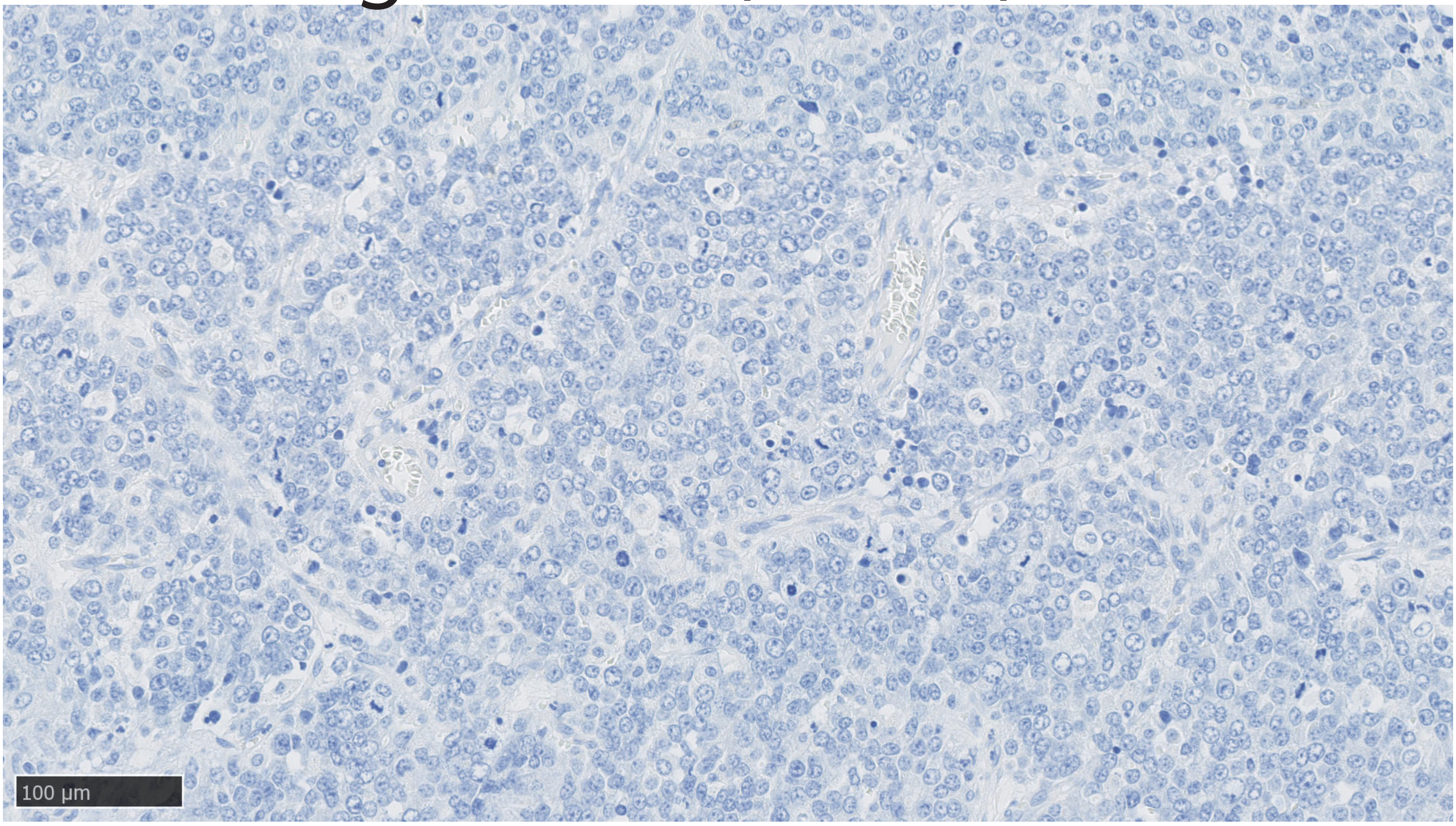

(G)
Cluster of differentiation 56 (CD56)

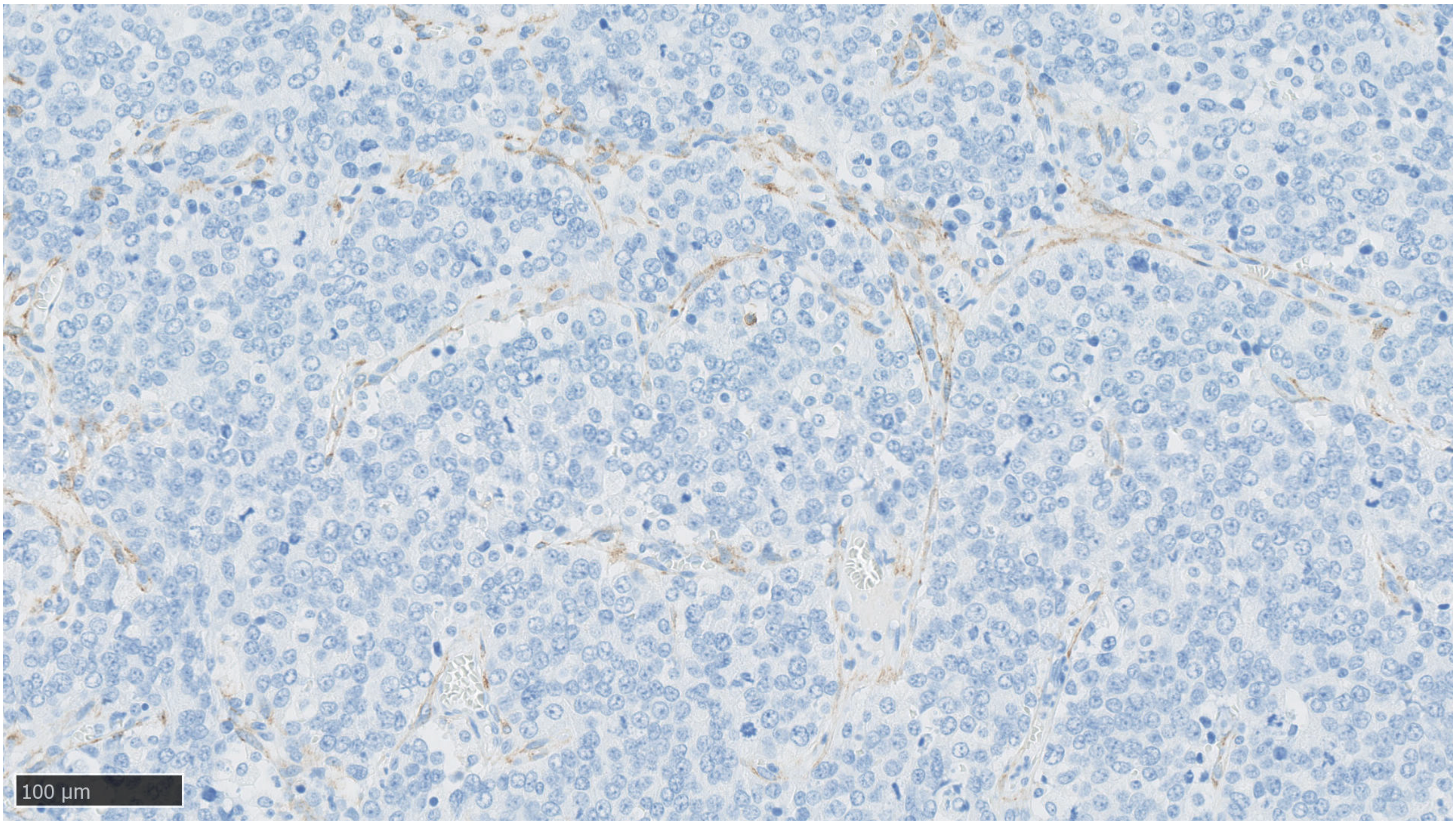

(H) Glioma-associated oncogene zinc finger 1 (GLI1)

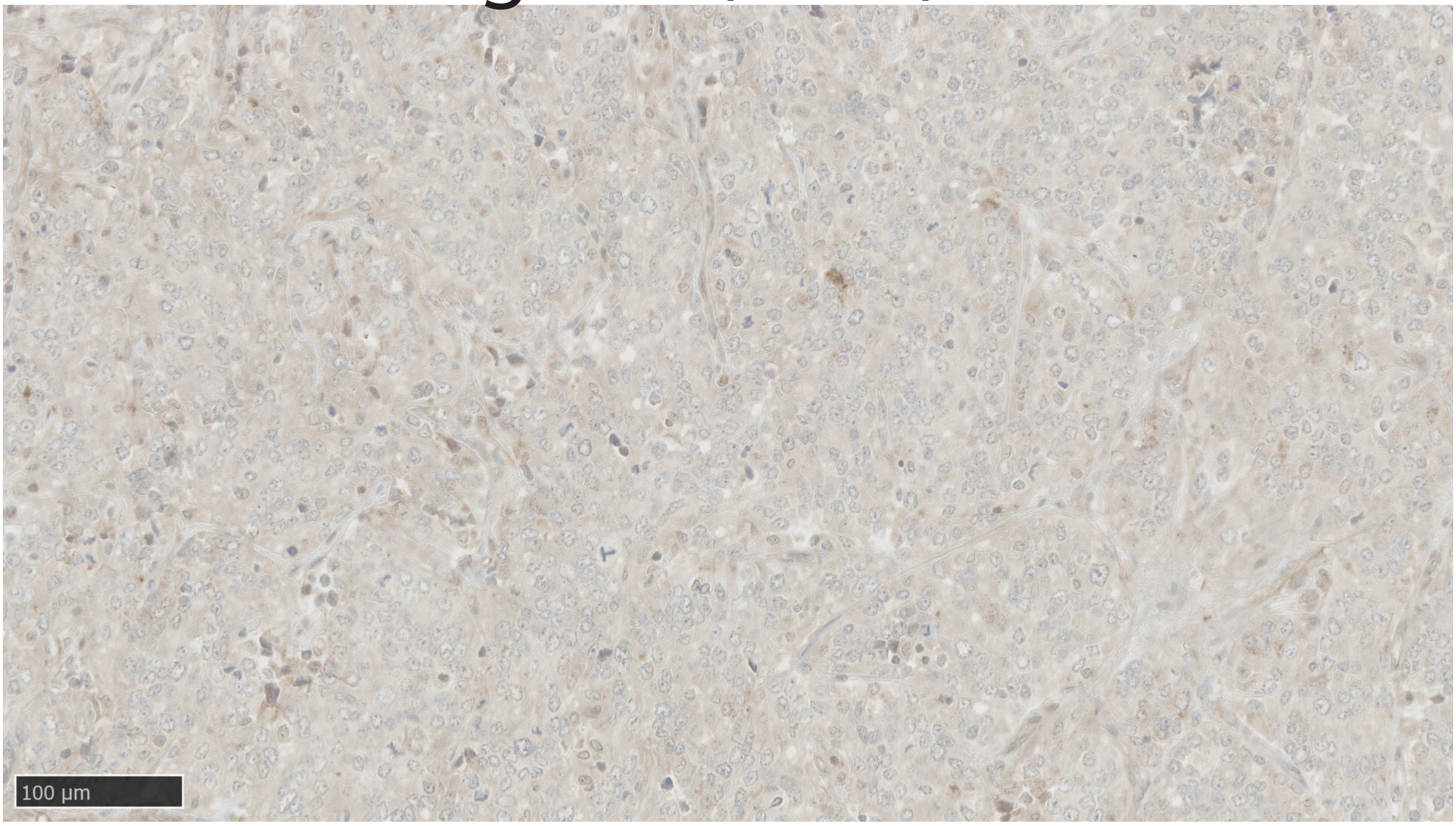

Supplement: Supplementary file 1 — Additional file1. Immunohistochemical staining of transurethral resection samples. (A) Tumor protein p53. (B) Retinoblastoma transcriptional corepressor. (C) Androgen receptor. (D) Prostate-specific antigen. (E) Synaptophysin. (F) Chromogranin A. (G) Cluster of differentiation 56. (H) Glioma-associated oncogene family zinc finger 1. The bars show 100μm. [file 12920_2022_1286_MOESM1_ESM.pdf]
